# Seizure after surgical treatment of chronic subdural hematoma—Associated factors and effect on outcome

**DOI:** 10.3389/fneur.2022.977329

**Published:** 2022-09-08

**Authors:** Hussam Hamou, Mohammed Alzaiyani, Tobias Rossmann, Rastislav Pjontek, Benedikt Kremer, Hasan Zaytoun, Hani Ridwan, Hans Clusmann, Anke Hoellig, Michael Veldeman

**Affiliations:** ^1^Department of Neurosurgery, RWTH Aachen University Hospital, Aachen, Germany; ^2^Department of Neurosurgery, Neuromed Campus, Kepler University Hospital, Linz, Austria; ^3^Department of Diagnostic and Interventional Neuroradiology, RWTH Aachen University, Aachen, Germany

**Keywords:** chronic subdural hematoma, seizure, clinical outcome, depressed brain volume, epilepsy, hematoma recurrence

## Abstract

**Introduction:**

Chronic subdural hematoma (cSDH) is becoming more prevalent due to population aging and the increasing use of antithrombotic drugs. Postoperative seizure in cSDH have a negative effect on outcome, and there currently no consensus regarding prophylactic anti-epileptic drug (AED) treatment. The objective of this study was to evaluate predisposing and triggering factors associated with postoperative epileptic seizure in patients with cSDH.

**Methods:**

All patients, who were surgically treated for cSDH in a single tertiary care center between 2015 and 2019, were considered for inclusion. Relevant patient- and hematoma-specific characteristics were retrospectively extracted from hospital records. Paroxysmal events categorized by the treating physician as suspected postoperative seizures were noted. The clinical outcome was extracted from the last available follow-up visit and classified according to the Glasgow outcome scale (GOS).

**Results:**

Of the included 349 patients, 54 (15.5%) developed suspected postoperative epileptic complications in the form of early seizure (≤ 7 days) in 11 patients (3.2%) and late seizure (>7 days) in 43 patients (12.3%). In the logistic regression analysis, solely depressed brain volume (supratentorial volume (ml) not filled with re-expanded brain) was independently associated with postoperative seizure (odds ratio [*OR*] 1.006, 95% *CI*: 1.001–1.011; *p* = 0.034). The occurrence of postoperative seizure (*OR* 6.210, 95% *CI*: 2.704–14.258; *p* < 0.001) and preoperative Markwalder grading (*OR* 2.919, 95% *CI*: 1.538–5.543; *p* = 0.001) were independently associated with unfavorable (GOS_1−3_) outcome.

**Conclusion:**

Larger postoperative depressed brain volume was the only factor independently associated with suspected postoperative seizure, and it could help identify a subgroup of patients with higher susceptibility to epileptic events. Based on our data, no formal recommendation can be made regarding the prophylactic use of anti-epileptic drugs. Nevertheless, the relative safety of new generation AEDs and the detrimental effect of postoperative seizure on outcome may justify its use in a selected patient population.

## Introduction

Chronic subdural hematoma (cSDH) constitutes one of the most prevalent disease entities requiring neurosurgical intervention. The estimated annual incidence varies from 7 to 30/100,000 in large population wide surveys and increases with age (1). The worldwide incidence of cSDH is rising, driven by population aging, increasing use of antithrombotic drugs (2), and the availability of cranial imaging (3). Though straightforward, surgical treatment is complicated by high hematoma recurrence rates, leading to retreatment and rising costs (4).

The exact pathophysiological chain of events in cSDH development is still incompletely understood. Minor head trauma with the collection of minimal acute subdural blood, either from a torn bridging vein or small contusion, has long been considered the initiator. An alternative hypothesis prioritizes the role of dural border cell separation and subdural hygroma formation as the initial stage. Hematomas typically enlarge over time, driven by localized inflammation and coagulopathy. Mass effect-induced symptoms usually lead to cranial computed tomography (CT) imaging and diagnosis. Fibroproliferation results in the membranization of the hematoma and the development of septa within. This most likely reflects an attempt toward natural repair and developmental stages in internal hematoma architecture have proved predictive of hematoma recurrence after treatment (5, 6).

The prevalence of postoperative seizure in cSDH varies between 0.67 and 32.0% (7, 8), and has negatively affected outcome in multiple observational series (9). Epileptogenesis in chronic subdural hematoma is incompletely understood and could result from mass-effect induced reduction in cerebral blood flow or from structural damage related to initial trauma. Breakdown hemoglobin products are highly epileptogenic and patients with mixed hematoma density on CT imaging—reflecting active hemo- and fibrinolysis—were found to have a higher risk of seizure (10). Hemoglobin degradation products are normally not in direct contact with the cerebral cortex, which is covered by visceral membrane in the more organized stages of chronic subdural hematoma. Membranes themselves and recurring microbleeds from neovasculature within could also provoke epileptic seizure, as proposed by Markwalder and Reulen (11). Prophylactic administration of anti-epileptic drugs (AED) in selected patients with cSDH has been debated, but no consensus has been reached to date. A 2013 Cochrane review addressing the issue could not provide any meaningful conclusion due to the methodological limitations of existing observational studies and the lack of randomized trials (12). The existing case series of patients with cSDH assessing postoperative seizures remain small and mostly pool pre- and postoperative epileptic complications together.

The primary objective of this study was to evaluate the role of predisposing patients and imaging characteristics associated with the occurrence of postoperative epileptic seizures. In more detail, we aim to evaluate whether hematoma architecture and membrane formation on CT imaging affects the risk of epileptic seizure after hematoma evacuation.

## Materials and methods

### Study population and design

All patients who were surgically treated for chronic subdural hematoma in a single tertiary care center between 2015 and 2019, were considered for inclusion. This study constitutes a subgroup analysis of previously published data (6). In the latter study, the influence of hematoma architecture on recurrence rates was assessed. The present study focuses on postoperative epileptic complications in relation to patient- and hematoma-specific characteristics. Our local ethics committee (EK399/20) approved the retrospective data collection, and the study was preregistered in the German Clinical Trials Register (DRKS00025280). Informed consent was waived due to the retrospective design. This manuscript is written in accordance with the STROBE statement for reporting observational studies. Data were collected retrospectively by screening of hospital records, extracting demographic data, clinical symptoms on admission, pre- and postoperative imaging characteristics, the occurrence of suspected epileptic seizures, hematoma recurrence, and clinical outcome. Consecutive patients were included when the diagnosis of cSDH led to surgical treatment either *via* burr hole, bone flap craniotomy, or twist drill craniostomy. In recurrent cases, the timing of seizure was related to the initial surgery and patients were only included once. Exclusion criteria were: (1) patients under the age of 18 years, (2) causal cranial procedures leading to cSDH, (3) intracranial hypotension that contributed to cSDH, (4) underlying non-iatrogenic coagulation disorders (e.g., hepatogenic coagulopathy), (5) prior treatment with AEDs due to seizure as a presenting symptom or preexisting epilepsy as comorbidity.

### Treatment algorithm

Our cSDH treatment algorithm has been previously described in detail (6). In summary, indication for the surgical evacuation of cSDH was based on the presence of neurological deficits, i.e., paresis, gait disturbance, or speech disorder. Asymptomatic hematomas were operated if mass effect was seen on imaging either in the form of ventricle compression and/or midline shift or sulcal effacement. Surgical technique varied and was up to surgeon's preference. The primary treatment option was a single-burr hole craniotomy with irrigation and placement of two subdural non-suction 12-French silicon drains in general anesthesia. In the case of intraoperative brain expansion and a tight subdural space, a single drain was inserted. Drains were removed after 1–3 days. Twist drill craniostomy in local anesthesia without irrigation was reserved for homogenous hematomas in frail patients. Bone-flap craniotomy was considered if the presence of hyperdense clot on CT imaging would suggest difficulty in complete hematoma evacuation *via* burr hole craniotomy. Visceral membranes were only opened if encapsulated deeper hematoma compartments were suspected. Otherwise, all membranous structures were left intact.

Patients were discharged with either complete symptom relief or post-operative imaging revealing no space-occupying residual hematoma. After discharge, follow-up imaging was routinely performed 14–28 days after surgery and continued until radiological resolution. Recurrence was defined as an increase in the volume of residual or newly formed hematoma with mass effect due to midline shift, sulcal effacement or new development or re-appearance of neurological symptoms (i.e., paresis, gait disturbance, and speech disorder) resulting in the need for reoperation.

### Radiological evaluation

**Figure 1:**
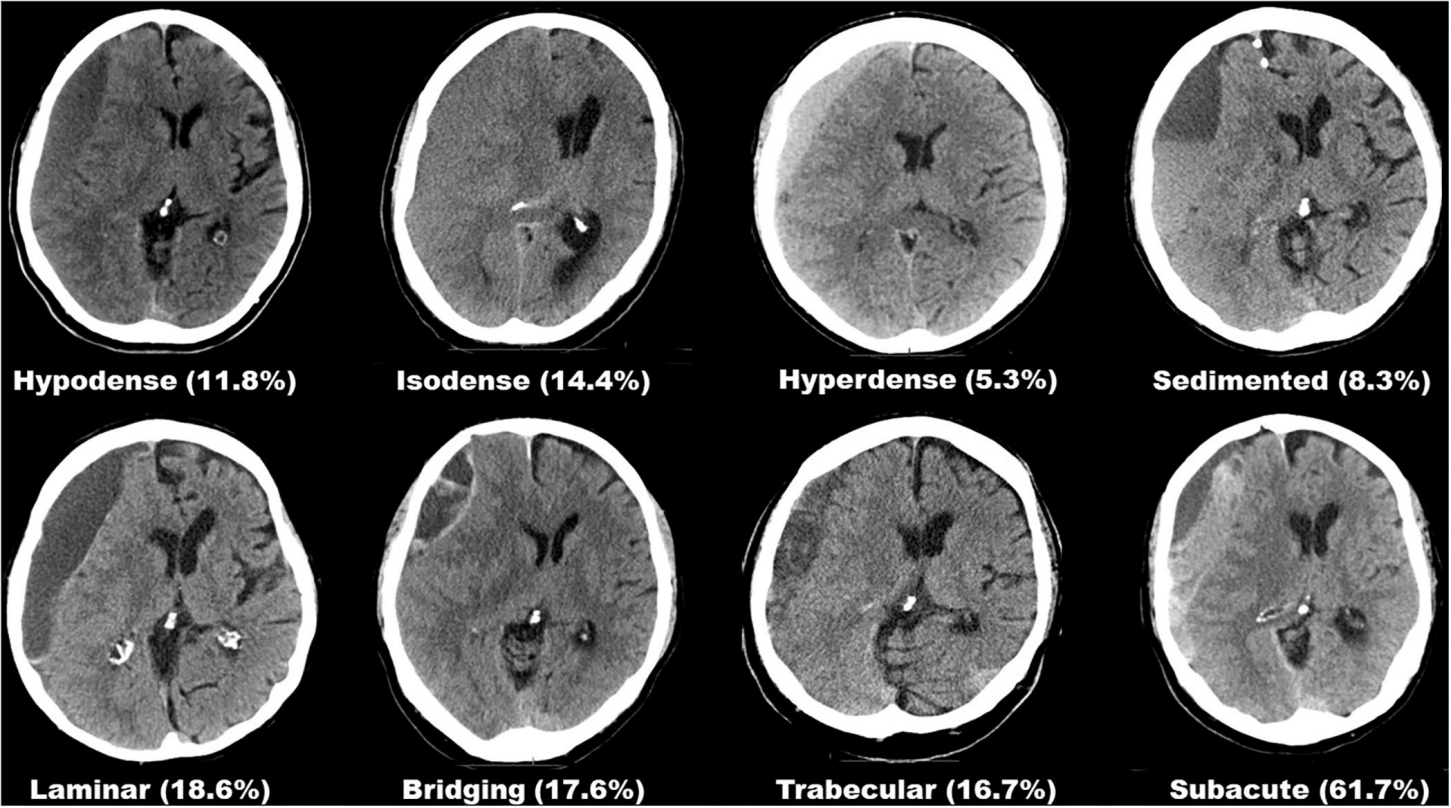
Overview of all eight hematoma subtypes based on internal architecture as seen in CT imaging and their respective rates of suspected seizure.

Preoperative hematoma dimensions were measured in axial CT images as: length along the longest axis (mm), width at its widest (mm), and volume (ml) were software assisted reconstructed (Brainlab, Munich, Germany). The presence of midline shift and its extension (mm) was noted. Two independent assessors (HH and MV), blinded to the outcome, classified each hematoma according to its structural appearance in CT imaging into one of eight subtypes, as described previously (6). All eight hematoma subtypes are depicted in Figure 1. Postoperative depressed brain volume (ml) was also software-assisted measured out (Brainlab, Munich, Germany) in post-operative imaging (13). Depressed brain volume includes all supratentorial intracranial volume not filled with re-expanded brain, e.g., residual hematoma, rinsing fluid, and air.

### Postoperative epileptic seizure

Paroxysmal events involving transient alterations in awareness, clonic movements, and/or aphasia/dysphasia without persisting symptoms after resolution, were classified as epileptic or not by the discretion of the treating physicians. Postoperative seizures were categorized according to the International League Against Epilepsy recommendations (14, 15) as either focal onset, generalized onset, or unknown/unclassified onset, based on clinical presentation. Electroencephalography (EEG) was reserved for cases refractory to the first line anticonvulsive treatment and not routinely available. Since non-epileptic intermittent symptoms cannot be distinguished from seizures, the terminology of suspected seizure is used throughout the text. The time point in relation to the day of surgery (day 0) was dichotomized into early (≤ 7 days) and late (>7 days) seizure onset. Patients with acute symptomatic seizure received lorazepam and, after the exclusion of treatable structural lesions in CT imaging (i.e., acute rebleeding), prophylactic anticonvulsive treatment was commenced in the form of levetiracetam as the first choice and lacosamide as the second choice, after evaluation of contraindications.

### Outcome evaluation

The clinical outcome was extracted from the last available documented follow-up visit and categorized according to the Glasgow outcome scale (GOS). The GOS was dichotomized into favorable (GOS_4−5_) or unfavorable (GOS_1−3_) outcome and the time-point of assessment was defined in days from diagnosis. The primary endpoint was defined as the occurrence of clinically diagnosed epileptic seizure after surgical hematoma evacuation. The secondary outcome was defined as the GOS after full hematoma resolution and/or upon last clinical follow-up.

### Statistical analysis

All data are presented as mean and standard deviation (SD) for normally, and as median and interquartile rage (IQR) (Q_1_-Q_3_) for non-normally distributed continues variables. Nominal and ordinal categorical data are presented as frequencies or proportions. Data were tested *via* the Shapiro-Wilk test for normality after which the appropriate statistical test was selected. Nominal data were tested with by means of the χ^2^ test and continuous data *via* the unpaired *t*-test or the Mann–Whitney *U*-test. A logistic regression model was built introducing all univariate factors with a *p*-value <0.15 in a single block (16). Associations are presented as odds ratios with 95% confidence intervals (CI). The Clopper–Pearson interval was used to calculate the binomial confidence intervals (CIs) around the incidence of suspected seizure. Before inclusion, variables were tested for outliers *via* plotting and multicollinearity was evaluated *via* the assessment of the Variance Inflation Factor with a cut-off of 2.5. Reported *p*-values are nominal without correction for multiple-hypothesis testing. Missing data were not imputed. All statistical analyses were performed using IBM SPSS Statistics 25 (SPSS Inc., Chicago, IL, USA). Statistical significance was defined as a two-sided *p* < 0.05.

## Results

Of 381 consecutive patients treated for chronic subdural hematoma, 20 patients were excluded due to preexisting epilepsy and 12 patients due to prophylactic treatment with AEDs after first-time seizure onset, which led to diagnosis of cSDH. None of these patients suffered postoperative suspected seizure. Of the remaining 349 patients, 54 (15.5%, 95% *CI*: 1.118–1.197) developed postoperative epileptic complications in the form of early seizure (≤ 7 days) in 11 patients (3.2%) and late seizure (>7 days) in 43 patients (12.3%). The median duration of follow-up was 79.0 days [33.0–152.0]. The most common type of acute symptomatic seizures was of focal onset and occurred in 37 (68.5%) patients. The generalized onset of seizure was observed in 9 (16.7%) patients and in 8 (14.8%) patients, seizure onset could not be classified. No patient progressed to status epilepticus.

Patients suffering postoperative seizures had on average thicker hematomas (23.9 mm ± 6.2) compared with patients not developing epileptic complications (22.2 mm ± 5.8; *p* = 0.005). Hematomas in patients with epileptic insults were on average 24.6 ml larger in volume (164.6 ml ± 67.2 *vs*. 140.0 ml ± 50.9; *p* = 0.005). However, the distribution of patients with midline shift and the extent thereof in preoperative imaging did not differ between patients developing postoperative seizures (9.6 mm ± 4.1) or not (9.0 mm ± 3.8; *p* = 0.500). Most patients were treated with burr hole craniotomy (247 patients, 65.3%). Bone flap craniotomy was performed in 10 (2.6%) patients, all suffering chronic subdural hematoma with signs of fresh blood content. Thereof, six patients developed postoperative epileptic seizures. Of all 54 patients with postoperative seizure, 35 (64.8%) developed favorable outcome, compared with 267 (90.5%) without epileptic complications (*p* < 0.001). Hematoma recurrence rates between patients with or without postoperative seizure were comparable (28.8 vs. 37.0%; *p* = 0.226). The comparison of relevant patient- and hematoma-specific characteristics and the complete outcome distribution according to the GOS is provided in Table 1. Factors affecting dichotomized clinical outcome are depicted in Table 2.

**Table 1:** Baseline characteristics, pre- and postoperative findings on CT imaging and outcome in chronic subdural hematoma patients with or without post-operative seizure.

	**All (*n =* 349)**	**No seizure (*n* = 295)**	**Post-operative seizure (*n* = 54)**	***p*-value**
**Demographics**				
Age—mean ± SD	75.6 ± 12.0	75.4 ± 11.8	76.3 ± 12.7	0.587
Sex—Female (%)/Male (%)	120 (34.4) / 229 (65.6)	105 (35.6) / 190 (64.4)	15 (27.8) / 39 (72.2)	0.266
**Initial presentation**				
Initial GCS—median [Q_1_ to Q_3_]	15 [14 to 15]	15 [14 to 15]	15 [14 to 15]	0.654
Markwalder grading—median [Q_1_ to Q_3_]	2 [1 to 2]	2 [1 to 2]	2 [1 to 2]	0.552
ASA classification	3 [2 to 3]	3 [2 to 3]	3 [2 to 3]	0.940
History of cranial trauma—no. (%)	252 (72.2)	221 (71.5)	41 (75.9)	0.507
**Preoperative deficit—no. (%)**				
Phatic disorder	71 (20.5)	60 (20.3)	11 (20.4)	0.956
Paresis	179 (51.2)	146 (49.5)	33 (61.1)	0.073
Gait disturbance	131 (37.5)	106 (35.9)	25 (46.3)	0.111
Personality changes/confusion	94 (26.9)	79 (27.8)	15 (27.8)	0.789
**Comorbidity—no. (%)**				
Hypertension	207 (59.3)	168 (56.9)	39 (72.2)	0.036
Atrial fibrillation	84 (24.1)	73 (24.7)	11 (20.4)	0.532
Coronary arterial disease	119 (34.1)	101 (34.2)	18 (33.3)	0.897
Diabetes	61 (17.5)	49 (16.6)	12 (22.2)	0.318
Cancerous disease	48 (13.8)	42 (14.2)	6 (11.1)	0.571
Alcohol abuse	15 (4.3)	13 (4.4)	2 (3.7)	0.832
History of DVT / PE	27 (7.7)	23 (7.8)	4 (7.4)	0.950
**Prior Medication—no. (%)**				
Antiplatelet	99 (28.4)	81 (27.5)	18 (33.3)	0.379
Anticoagulants	83 (23.8)	70 (23.7)	13 24.1)	0.956
Of which NOAC	22 (6.3)	18 (6.1)	4 (7.4)	0.717
**Hematoma imaging characteristics**				
Left side—no. (%)	118 (33.8)	104 (35.3)	14 (25.9)	0.268
Bilateral—no. (%)	85 (24.4)	73 (24.7)	12 (22.2)	0.691
Width (mm)—mean ± SD	22.5 ± 5.9	22.3 ± 5.8	23.9 ± 6.2	**0.005**
Length (mm)—mean ± SD	121.9 ± 22.4	121.1 ± 23.0	126.7 ± 17.7	0.178
Volume (ml)—mean ± SD	143.3 ± 53.9	140.0 ± 50.9	164.6 ± 67.2	**0.005**
Midline Shift—no. (%)	235 (67.3)	200 (67.8)	35 (64.4)	0.668
MLS (*n* = 235) (mm)—mean ± SD	9.1 ± 3.8	9.0 ± 3.8	9.6 ± 4.1	0.500
**Internal Architecture—no. (%)**				
Homogenous hypodense	68 (19.5)	60 (20.3)	8 (14.8)	0.346
Homogenous isodense	83 (23.8)	71 (24.1)	12 (22.2)	0.770
Homogenous hyperdense	19 (5.4)	18 (6.1)	1 (1.9)	0.206
Sedimented	24 (6.9)	22 (7.5)	2 (3.7)	0.316
Laminar	43 (12.3)	35 (11.9)	8 (14.8)	0.544
Bridging	34 (9.7)	28 (9.5)	6 (11.1)	0.712
Trabecular	54 (15.5)	45 (15.3)	9 (16.7)	0.792
With signs of fresh blood component	24 (6.9)	16 (5.4)	8 (14.8)	**0.012**
Membranous hematoma	131 (37.5)	108 (36.6)	23 (42.6)	0.213
**Surgical treatment—no. (%)**				**0.001**
Burr hole	234 (67.0)	196 (66.4)	38 (70.4)	
Twist drill	106 (30.4)	95 (32.2)	11 (20.4)	
Bone flap	9 (2.6)	5 (1.7)	5 (9.3)	
**Postoperative outcome**				
Depressed brain volume (ml)—median [IQR] (*n* = 275)	78.0 [47.6 to 117.8]	76.9 [47.9 to 107.8]	102.0 [44.3 to 175.0]	0.053
Need for ICU surveillance	66 (18.9)	49 (16.6)	17 (31.5)	**0.010**
ICU LOS (*n* = 80)—median [Q1 to Q3]	2 [1.0 to 7.0]	2 [1.0 to 5.0]	7 [1.0 to 11.5]	**0.030**
GOS-−12 months—no. (%)				**<0.001**
Good recovery	251 (71.9)	226 (76.6)	25 (46.3)	
Moderate disability	51 (14.6)	41 (13.9)	10 (18.5)	
Severe disability	29 (8.3)	16 (5.4)	13 (24.1)	
Vegetative state	2 (0.6)	2 (0.7)	0	
Dead	16 (4.6)	10 (3.4)	6 (11.1)	
Favorable outcome	302 (86.5)	267 (90.5)	35 (64.8)	**<0.001**
**Recurrence-no. (%)**	105 (30.1)	85 (28.8)	20 (37.0)	0.226

ASA, American society of anesthesiologists—physical status classification; DVT, deep vein thrombosis; GCS, Glasgow coma scale; GOS, Glasgow outcome scale; ICU, intensive care unit; Q1 to Q3, interquartile range; LOS, length of stay; MLS, midline shift; NOAC, new oral anticoagulants; PE, pulmonary embolism; SD, standard deviation. Significant values below *p* < 0.05 are marked in bold.

**Table 2:** Analysis of factors effecting outcome after the surgical treatment of chronic subdural hematoma.

	**Favorable outcome (*n* = 302)**	**Unfavorable outcome (*n* = 47)**	***p*-value**
**Demographics**			
Age—mean ± SD	74.8 ± 12.2	80.6 ± 8.7	**0.002**
Sex—Female (%)/Male (%)	106 (35.1)/196 (64.9)	14 (29.8)/33 (70.2)	0.476
**Initial presentation**			
Initial GCS—median [Q1 to Q3]	15 [15 to 15]	14 [13 to 15]	**<0.001**
Markwalder grading—median [Q1 to Q3]	1 [1 to 2]	2 [2 to 2]	**<0.001**
ASA classification	3 [2 to 3]	3 [3 to 3]	**0.035**
History of cranial trauma—no. (%)	218 (72.2)	34 (72.3)	0.982
**Preoperative deficit—no. (%)**			
Phatic disorder	57 (18.9)	14 (29.8)	0.063
Paresis	145 (48.0)	34 (72.3)	0.001
Gait disturbance	118 (39.1)	13 (27.7)	0.206
Personality changes/confusion	81 (26.8)	13 (27.7)	0.801
**Co-morbidity—no. (%)**			
Hypertension	172 (57.0)	35 (74.5)	**0.023**
Atrial fibrillation	67 (22.2)	17 (36.2)	**0.029**
Coronary arterial disease	97 (32.1)	22 (46.8)	**0.049**
Diabetes	54 (17.9)	7 (14.9)	0.616
History of cancerous disease	37 (12.3)	11 (23.4)	**0.033**
Alcohol abuse	12 (4.0)	3 (6.5)	0.428
History of DVT/PE	22 (7.3)	5 (10.6)	0.397
**Prior Medication—no. (%)**			
Antiplatelet	86 (28.5)	13 (27.7)	0.908
Anticoagulants	62 (20.5)	21 (44.7)	**<0.001**
Preoperative INR	1.17 ± 0.61	1.27 ± 0.52	**0.002**
Of which NOAC	17 (5.6)	5 (10.6)	0.189
**Hematoma imaging characteristics**			
Left side—no. (%)	102 (33.8)	16 (34.0)	0.493
Bilateral—no. (%)	79 (26.2)	9 (19.1)	0.371
Width (mm)—mean ± SD	21.6 ± 5.6	23.2 ± 7.6	0.220
Length (mm)—mean ± SD	120.1 ± 21.5	126.0 ± 21.0	**0.002**
Volume (ml)—mean ± SD	145.1 ± 57.9	138.7 ± 63.2	0.755
Midline Shift—no. (%)	204 (67.5)	31 (66.0)	0.829
MLS (*n* = 235) (mm)—mean ± SD	9.0 ± 3.9	9.9 ± 5.1	0.246
**Internal Architecture—no. (%)**			
Homogenous hypodense	62 (20.5)	6 (12.8)	0.211
Homogenous isodense	75 (24.8)	8 (17.0)	0.242
Homogenous hyperdense	16 (5.3)	3 (6.4)	0.760
Sedimented	21(7.0)	3 (6.4)	0.886
Laminar	34 (11.3)	9 (19.1)	0.126
Bridging	30 (9.9)	4 (8.5)	0.760
Trabecular	45 (14.9)	9 (19.1)	0.454
With signs of fresh blood component	19 (6.3)	5 (10.6)	0.273
Membranous hematoma	109 (36.1)	22 (46.8)	0.158
**Postoperative outcome**			
Depressed brain volume (ml)—median [Q1 to Q3] (*n* = 275)	78.5 [47.9 to 114.5]	90.0 [45.0 to 165.4]	0.361
Recurrence- no. (%)	95 (31.5)	10 (21.3)	0.157

ASA, American society of anesthesiologists—physical status classification; DVT, deep vein thrombosis; GCS, Glasgow coma scale; GOS, Glasgow outcome scale; Q1 to Q3, interquartile range; MLS, midline shift; NOAC, new oral anticoagulants; PE, pulmonary embolism; SD, standard deviation. Significant values below *p* < 0.05 are marked in bold.

### Factors associated with epileptic complications

Postoperative depressed brain volume could only be calculated in 275 (78.8%) patients due to the unavailability of postoperative imaging studies in the remaining patients. A binomial logistic regression model was built to identify factors associated with postoperative epileptic seizure. Based on univariate analysis, six factors were identified to include into the model: the presence of paresis or gait disturbance upon initial presentation, hypertension as comorbidity, hematoma width, chronic subdural hematoma with signs of fresh blood content, and postoperative depressed brain volume. Hematoma volume was excluded based on collinearity with hematoma width. The logistic regression model was statistically significant χ^2^(6) = 16.129, *p* = 0.013. The model explained 13.2% (Nagelkerke *R*^2^) of the variance in seizure occurrence and correctly classified 81.2% of cases. Of the six predictor variables, only depressed brain volume was independently associated with postoperative seizure (*OR* 1.006, 95% *CI*: 1.001–1.011; *p* = 0.034), as shown in Table 3.

**Table 3:** Results of the logistic regression analysis of factors effecting postoperative seizure and dichotomized outcome.

	**No seizures (*n* = 294)**	**Postoperative seizure (*n* = 54)**	**Odds ratio**	**Confidence interval**	***p*-value**
Paresis—no. (%)	146 (49.5)	33 (61.1)	1.216	0.533 to 2.772	0.642
Gait disturbance—no. (%)	106 (35.9)	25 (46.3)	1.440	0.666 to 3.112	0.354
Hypertension—no. (%)	168 (56.9)	39 (72.2)	1.683	0.709 to 3.998	0.238
Width (mm)—mean ± SD	22.3 ± 5.8	23.9 ± 6.2	1.045	0.974 to 1.120	0.219
Subacute	16 (5.4)	8 (14.8)	2.603	0.698 to 9.705	0.154
Depressed brain volume (ml)—median [IQR]	76.9 [47.9 to 107.8]	102.0 [44.3 to 175.0]	1.006	1.001 to 1.011	**0.034**
	**Favorable outcome (*****n*** = **302)**	**Unfavorable outcome (*****n*** = **47)**	**Odds Ratio**	**Confidence Interval**	***p*** **-value**
Post-operative seizure—no. (%)	35 (11.6)	19 40.4)	6.210	2.704 to 14.258	**<0.001**
Age—mean ± SD	74.8 ± 12.2	80.6 ± 8.7	1.032	0.987 to 1.078	0.172
Markwalder grading—median [IQR]	1 [1 to 2]	2 [2 to 2]	2.919	1.538 to 5.543	**0.001**
Phatic disorder—no. (%)	57 (18.9)	14 (29.8)	1.376	0.604 to 3.134	0.447
Paresis—no. (%)	145 (48.0)	34 (72.3)	1.451	0.609 to 3.456	0.401
Hypertension—no. (%)	172 (57.0)	35 (74.5)	1.009	0.429 to 2.371	0.984
Atrial fibrillation—no. (%)	67 (22.2)	17 (36.2)	1.495	0.587 to 3.806	0.399
Coronary arterial disease—no. (%)	97 (32.1)	22 (46.8)	1.515	0.637 to 3.605	0.348
History of cancerous disease—no. (%)	37 (12.3)	11 (23.4)	2.461	0.976 to 6.209	0.056
Preoperative INR	1.17 ± 0.61	1.27 ± 0.52	1.069	0.566 to 2.019	0.836
Length (mm)—mean ± SD	120.1 ± 21.5	126.0 ± 21.0	1.012	0.994 to 1.031	0.203

INR, international normalized ration; Q_1_ to Q_3_, interquartile range; mm, millimeter; ml, milliliter; MLS, midline shift; SD, standard deviation.

### Effects of epileptic complications on outcome

A second binomial logistic regression model was built to identify factors associated with an unfavorable outcome. Based on the univariate analysis, 11 factors were identified to be included into the model: the occurrence of postoperative seizure, age, Markwalder grading, speech disorder and paresis as presenting symptom, hypertension, atrial fibrillation and coronary arterial disease as comorbidities, history of cancerous disease, preoperative INR, and hematoma length. ASA grading and initial GCS were omitted based on the collinearity with Markwalder grading. The logistic regression model proved significant with χ^2^(11) = 60.475, *p* < 0.001. The model explained 30.1% (Nagelkerke *R*^2^) of the variance in an unfavorable outcome and correctly classified 88.9% of the cases. Of the 11 predictor variables, only postoperative seizure (*OR* 6.210, 95% *CI*: 2.704–14.258; *p* < 0.001) and preoperative Markwalder grading (*OR* 2.919, 95% *CI*: 1.538–5.543; *p* = 0.001) were independently associated with an unfavorable outcome (as shown in Table 3). Postoperative seizure doubled the rate of patients requiring intensive care unit (ICU) surveillance (31.5 *vs*. 16.6%; *p* = 0.010) and median ICU length of stay was longer for these patients (7 days [1.0–11.5] *vs*. 2 days [1.0–5.0]; *p* = 0.030) (Table 1).

## Discussion

**Figure 2:**
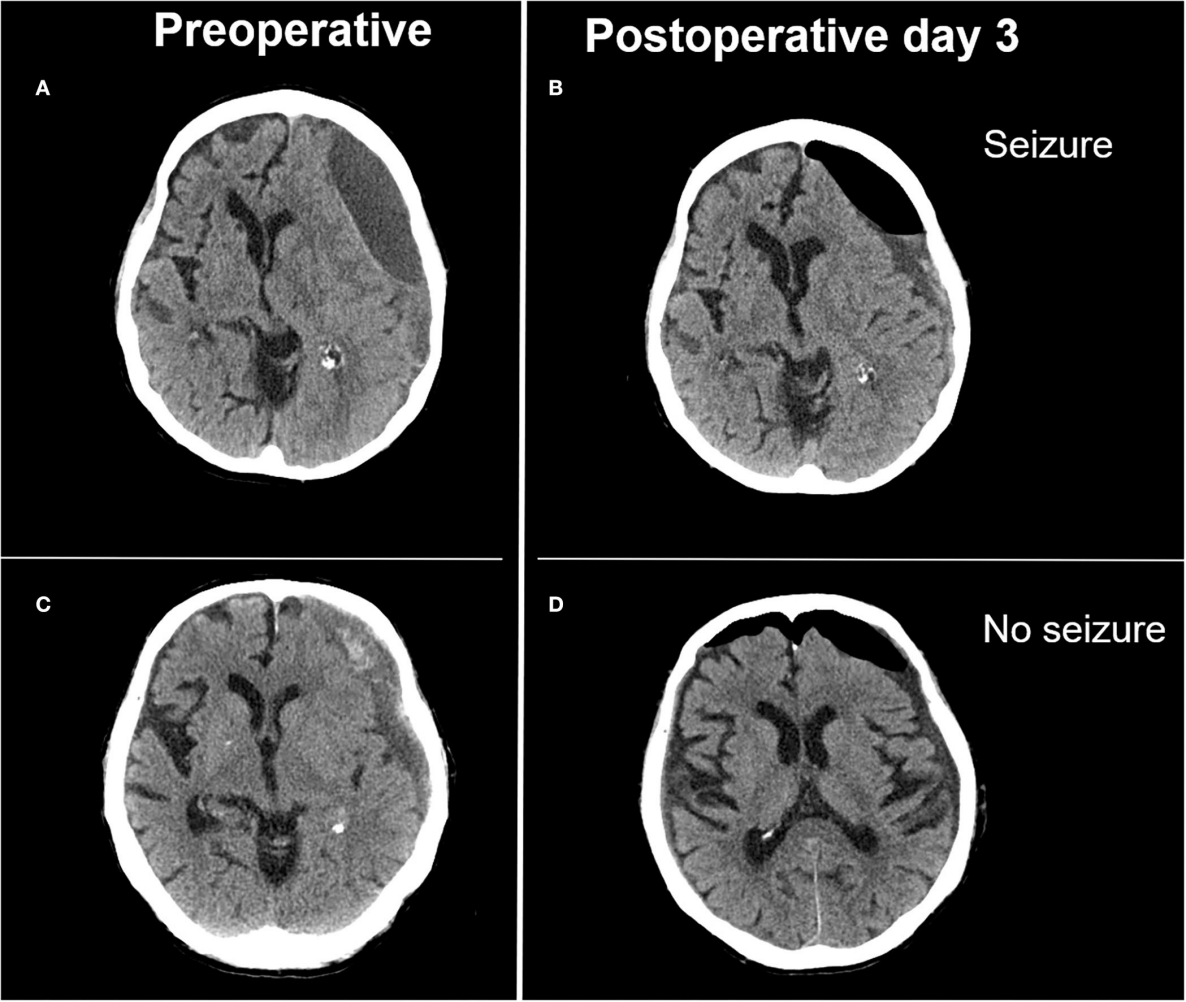
Illustrative case of extensive depressed brain volume leading to postoperative seizure and adequate postoperative brain expansion not associated with postoperative seizure. **(A)** Preoperative image of a laminar type hematoma demonstrating limited brain re-expansion **(B)** after hematoma evacuation. Patient presented with focal seizures on postoperative day 3. **(C)** Preoperative image of a trabecular type hematoma with adequate brain re-expansion **(D)** and no postoperative seizure activity.

Seizures after treatment for chronic subdural hematoma considerably complicate hospital stays and can even necessitate intensive care observation and treatment. In this consecutive 4 years series of patients surgically treated for cSDH, the observed rate of epileptic seizures of 15.5% lies at the upper margins of incidences previously described in a similar series (9, 17). Only postoperative depressed brain volume proved independently associated with seizure occurrence (Figure 2). An epileptic seizure was, alongside initial clinical state, a significant predictor of clinical outcome and increased the rate of unfavorable outcome as measured *via* the Glasgow outcome scale.

**Figure 3:**
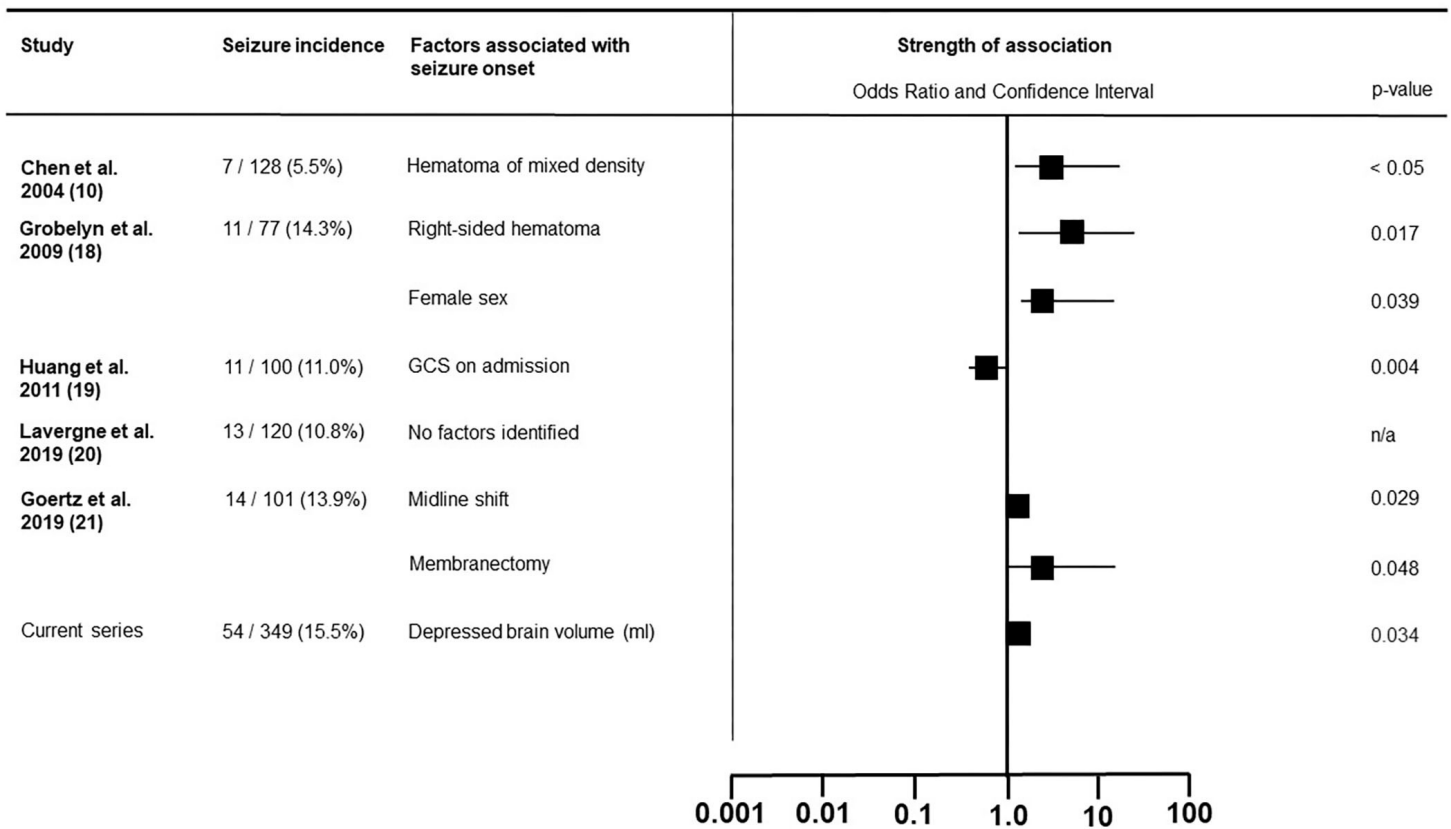
Literature overview of the factors associated with postoperative epileptic seizure in patients surgically treated for chronic subdural hematoma.

We hypothesized that some architectural hematoma subtypes would prove more epileptogenic than others. This either due to the contact of hemoglobin breakdown products with cortical surface in earlier stages of hematoma development, or direct irritation by the hematoma's visceral membrane once they have been formed. This latter concept was debuted by Markwalder and Reulen (11) who postulated the epileptogenicity of membranes overlaying the cortical surface due to either direct irritation or microbleeding. We focused on postoperative epileptic events, as these are potentially avoidable by prophylactic anticonvulsive treatment. We have summarized all existing published series, which assessed factors effecting postoperative seizures in Figure 3 (10, 18–21). In contrast with our results, Chen et al. described how mixed density hematomas—corresponding to the sedimented type in Nakaguchi et al.'s (5) and our classification (6)—were associated with a higher rate of postoperative seizure, whereas in our cohort this subtype presented with the lower rates of seizure compared to other architectural subtypes (10). In our series, the chronic subdural hematomas with signs of fresh blood, demonstrated the highest epileptic complication rate with one-third of patients suffering postoperative seizure. However, possibly due to low prevalence, the presence of an acute blood component was not an independently associated with seizure in our regression model. We believe hematoma membranes alone have no influence on the development of seizures. In our cohort, membranous hematoma subtypes did not present with higher pre- or postoperative seizure rates. Calcified membranes in cSDH are known to be epileptogenic, however, this entity was not encountered in this series (22).

In a series of 101 patients with cSDH, Goertz et al. identified membranectomy to be associated with postoperative epileptic seizure (21). This much more traumatic procedure compared with active rinsing and passive drainage has come into disfavor in more contemporary series as no true benefit in respect to the recurrence rate, has been observed (23).

Factors contributing to seizure onset could be roughly divided into either predisposing factors lowering seizure threshold or factors directly provoking seizures. Increasing age, brain atrophy, and prior head trauma increase susceptibility to epileptic seizure, whereas cortical manipulation, abrupt pressure relieve during surgery, and gliosis could directly induce epileptic activity. Early postoperative depressed brain volume was identified in our series as the sole predictor of postoperative seizure and reflects the lack of brain re-expansion capacity. It might be that this parameter was identified because it benefits from both entailing information on predisposing factors, i.e., atrophy as well as on the brain's response to surgical treatment and sudden relieve of pressure. However, use of this parameter to identify patients eligible for prophylactic AED administration would require routing postoperative imaging.

Relevant differences in postoperative seizure rates were observed between three surgical procedures. The highest seizure rate was seen after craniotomy and the lowest after the twist drill craniostomy. As the choice of procedure was based on radiological presentation, it was not possible to independently assess the causal relation between the type of procedure and seizure risk. Especially, cortical irritation by drain placement and removal, might prove an epileptogenic trigger in predisposed brain. On the other hand, subperiostal drain placement did not present with lower seizure rates compared with subdural placement in a randomized controlled trial (24). Drain misplacement can contribute to epileptogenisis and misplacement rates up to 15.8% have been reported (25). This factor could not be assessed in this series because routine imaging to exclude drain misplacement was not part of our institutional algorithm.

The paroxysmal nature of seizures creates significant limitations in diagnostic precision. The gold-standard ictal electrophysiological corroboration is unrealistic in daily clinical practice. Even if performed, false-negative EEGs remain a common problem due to interictal timing or already effective treatment. Possible overdiagnosis of epileptic seizures is possible due to this lack of diagnostic standardization. In addition, the clinical threshold to commence AED treatment might have been lowered since the availability of new generation anticonvulsants with a narrower spectrum of side effects. Nonetheless, side effects exist and though being less obvious, neurocognitive impairment and psychiatric symptoms might prove very debilitating. The problem of overdiagnosis has been addressed by the introduction of the concept of transient neurological deficit, as an all-encompassing entity in cSDH, without etiological boundaries. In their series of 1,307 patients with cSDH, Blaauw et al. identified 113 patients with transient neurological deficit (26). Somewhat less than half were classified as epileptic seizures by treating physicians, based on clinical presentation. We acknowledge the usefulness of this entity in preventing overdiagnosis of epileptic seizure but both pathophysiological explanation and treatment recommendations are missing. In our opinion, the risk of repeated seizures possibly necessitation intensive care surveillance, outweighs the undesirable side effects of new generation anticonvulsants.

Cortical spreading depolarization might be an alternative cause of transient neurological symptoms in patients suffering from cSDH (27, 28). Cortical spreading depolarization was detected in 6 (15%) out of 40 patients with cSDH, as measured *via* subdural strip electrodes (29). Even if events like this could contribute to transient neurological symptoms, it remains unclear how to therapeutically respond (30).

Eventually, the central question remains whether it is useful to select patients for prophylactic anticonvulsant treatment. A recent meta-analysis fusing data of six observational studies was not able to identify any reduction of seizure incidence by the administration of prophylactic antiepileptics (9). An elaborate systematic review focusing on posttraumatic seizures, such as chronic subdural hematoma, was inconclusive as to which patients should be prophylactically treated (17). Unfortunately, despite being larger than most existing series in these reviews, our study is not able to shed more light on the issue. Interestingly, in contrast to other observational series, no preoperative hematoma characteristics, such as size, internal architecture, or severity of initial clinical symptoms were associated with seizure onset.

This study is subjected to reporting bias as seizure onset and symptoms were retrospectively retrieved from electronic patient files. Continuous EEG monitoring, as applied in some centers, was not performed in this series, and therefore non-convulsive seizure could not be detected and non-epileptic paroxysmal events cannot be excluded. The non-epileptic, stereotypical, and intermittent symptom score (NESIS) scoring system has been developed to quantify characteristics of paroxysmal events unspecific to epileptic seizure (31). We were not able to reconstruct the score and exclude potential non-epileptic events, because documentation of episodes was often too vague, or onset was simply not witnessed by medical staff. This could have led to seizure overdiagnosis, whereas, on the other hand, post-operative seizures after discharge treated in other neurological units/hospitals might not have been reported to us thus may be missing in our data. Moreover, we were not able to assess which patients progressed to chronic epilepsy as most series, similar to ours, applied treatment after the initial seizure. The natural course of disease without AEDs remains therefore unclear. Anticonvulsants are usually progressively weaned once cSDH has demonstrated full resolution, but the timing of discontinuation remains controversial. Based on the retrospective cohort analysis by Huang et al., prophylactic treatment should not exceed 2 years as all seizures in their cohort occurred earlier (19). Clinical outcome was also retrospectively collected resulting in a high variability in follow-up. By the assessment of patients after different periods of recovery, clinical outcome is heavily biased. Finally, because not all patients received early post-operative CT-imaging, depressed brain volume could only be calculated in 275 (78.8%) patients, introducing selection bias which cannot be corrected for.

## Conclusion

No formal recommendation can be made regarding the prophylactic use of anti-epileptic drugs, based on our data. Nevertheless, the relative safety of new generation AEDs and the detrimental effect of postoperative seizure on the need for ICU surveillance and outcome may justify its use in a selected patient's population. Based on our cohort, larger postoperative depressed brain volume constituted the only factor independently associated with postoperative seizure onset possibly because it entails information on predisposing factors as well as patient's response to surgery. Larger depressed brain volume could help identify a subgroup of patients with higher susceptibility for postoperative epileptic events.

## Data availability statement

The raw data supporting the conclusions of this article can be made available by the authors to any qualified researcher.

## Ethics statement

The studies involving human participants were reviewed and approved by Ethics Committee of the Medical Faculty of the RWTH Aachen University (EK399/20). Written informed consent for participation was not required for this study in accordance with the national legislation and the institutional requirements.

## Author contributions

HH and MV developed the design and conception of this study and performed the statistical analysis. HH, MA, RP, BK, HZ, HR, AH, and MV were involved in the acquisition of data. HH, TR, and MV drafted the manuscript. All authors were involved in the interpretation of data. The final manuscript was critically revised and approved by all authors.

## Conflict of interest

The authors declare that the research was conducted in the absence of any commercial or financial relationships that could be construed as a potential conflict of interest.

## Publisher's note

All claims expressed in this article are solely those of the authors and do not necessarily represent those of their affiliated organizations, or those of the publisher, the editors and the reviewers. Any product that may be evaluated in this article, or claim that may be made by its manufacturer, is not guaranteed or endorsed by the publisher.

## Abbreviations

AED: anti-epileptic drugs
ASA: American society of anesthesiologists—physical status classification
CI: confidence interval
cSDH: chronic subdural hematoma
CT: computed tomography
GCS: Glasgow coma scale
INR: international normalized ratio
mm: millimeter
ml: milliliter
n/a: not available
NOAC: new oral anticoagulants
OR: odds ratio
Q_1_ to Q_3_: first quartile to third quartile
SD: standard deviation.
